# Utility of the joint index in newborn congenital heart disease screening

**DOI:** 10.1038/s41598-025-10450-y

**Published:** 2025-07-19

**Authors:** Mengwen Li, Dan He, Tingting Peng, Rui Liu, Binzhi Tang

**Affiliations:** 1 Department of Pediatrics, Sichuan Provincial People’s Hospital, University of Electronic Science and Technology of China, Chengdu, China; 2 Department of Cardiology, Chengdu Jinniu District People’s Hospital (Sichuan Provincial People’s Hospital Jinniu Hospital), Chengdu, Sichuan Province China

**Keywords:** Newborn, Congenital heart disease, Heart murmur, Pulse oximetry, Screening, Cardiology, Medical research, Signs and symptoms, Diseases, Cardiovascular diseases

## Abstract

To examine the value of“joint index”in the early and rapid screening of congenital heart disease (CHD) in newborns. The study selected live-born neonates who were delivered in the Department of Obstetrics and Gynecology at Sichuan Provincial People’s Hospital from January 1, 2023 to December 31, 2023. Cardiac auscultation and pulse oximetry were performed at 6 h to 72 h after birth. To further elucidate the diagnosis and to analyze and compare the value of cardiac murmur, pulse oximetry, and the combination of cardiac murmur and pulse oximetry for the early diagnosis of CHD, cardiac ultrasonography was performed on newborns with positive screening for either cardiac murmur or pulse oximetry. The value of heart murmur, pulse oximetry, and their combination was assessed in the early diagnosis of CHD. A total of 3037 newborns were screened following the inclusion and exclusion criteria. Of them, a total of 304 positive cases were included in the present study and were divided into 3 groups: 142 cases in the heart murmur group, 99 cases in the oxygen saturation group, and 304 cases in the combined group of those who were positive for both heart murmur and/or oxygen saturation. A total of 215 cases of CHD were diagnosed via cardiac ultrasound in screen-positive newborns, including 26 cases of severe CHD. The top three types of CHD were atrial septal defect (33.95%), ventricular septal defect (26.05%), and arterial duct failure (20.47%). The diagnostic sensitivity, accuracy, and negative predictive value of the combined group were 100%, 97.07%, and 100%, respectively. These values were significantly higher than those of the heart murmur group and the oxygen saturation group (*P* < 0.01), and the overall difference among the three groups in terms of the positive predictive value was not statistically significant (*P* > 0.05). However, in the two-by-two comparisons within the groups, the positive predictive value of the combined group was greater than that of the oxygen saturation group, and the difference was statistically significant (70.72% vs. 50.89%; *P* < 0.05). The specificity of the combined group was 96.85%, which was lower than that of the murmur and oxygenation groups (98.37% vs. 98.55%; *P* < 0.01). However, considering the detection of severe CHD, a total of 14 cases of severe CHD were detected in patients with positive cardiac murmur and oxygenation in the combined group (53.83%), which was significantly higher than that in the murmur group (11.54%) and oxygenation group (34.62%, *P* < 0.01). The newborns can be screened for CHD using heart murmur auscultation combined with pulse oximetry, which is a convenient, sensitive, and accurate method. It can be utilized as an important tool for early clinical screening for CHD.

## Introduction

Birth defects are the primary cause of early miscarriage, stillbirth, perinatal mortality, and child disability. According to statistics, 800–1.2 million children are born with defects annually in China, with a total incidence of birth defects of 5.6%, and a defective child is born approximately every 30 s^1,2^, and the incidence of congenital heart disease (CHD) is the leading birth defect. CHD is a congenital malformation occurring owing to the abnormal structural development of the heart or large blood vessels during fetal life, posing a serious threat to children’s health and bringing a huge economic burden to society and families. As per the current statistics, its detection rate is approximately 2.9–16%^3,4^. However, not all patients with CHD can be diagnosed at an early stage because of the geographical and economic conditions, so the actual prevalence rate should be higher^5^. Additionally, with the full liberalization of the “three-child policy” in China, the prevention and treatment of CHD are facing more serious challenges.

The clinical screening for CHD primarily relies on medical history, physical examination, electrocardiography, and echocardiography. Among these, echocardiography is imperative in the clinical diagnosis of CHD; however, advocating it is difficult in primary hospitals owing to the high requirements for equipment and operators. Furthermore, it is not practical to conduct echocardiographic examinations on all newborns during this period.The traditional clinical screening method for neonatal CHD is auscultation of heart murmurs, which is simple and easy but more subjective. Because the circulatory system of newborns is in the stage of rapid adjustment and adaptation after birth, most of the heart murmurs are physiological; therefore, they must be combined with other means of judgment. Pulse oximetry is known as a “milestone” in the history of CHD screening and is a non-invasive instrument that indirectly monitors hemoglobin oxygenation in vivo. The American Academy of Pediatrics in a policy statement in 2012, advocated the incorporation of sophisticated, non-invasive, and painless pulse oxygen saturation SpO2 monitoring into routine neonatal monitoring to enhance the detection rate of severe CHD^6^. However, it cannot precisely reveal the actual oxygenation condition when there are circulatory abnormalities such as skin yellowing and shock. Moreover, detecting CHD without hypoxemia is not feasible by relying on SpO2 alone, so there is a probability of underdiagnosis. Therefore, a combination of heart murmur auscultation and SpO2 screening seems to be an effective and feasible means to improve the detection rate of neonatal CHD and avoid the disadvantages of either heart murmur auscultation alone or SpO2 screening alone.

In 2016, Professor Huang Guoying’s team proposed combining heart murmur and percutaneous oxygen saturation (SpO_2_ ≤ 95%) as screening indicators, establishing a ‘dual-indicator’ screening system (sensitivity 93.2%, specificity 97.1%)^7^. This approach was incorporated into the national neonatal congenital heart disease screening system in 2018^8^ This has been gradually promoted nationwide and is highly significant for the early detection and diagnosis of neonatal CHD in China. However, large-scale research data are lacking, and further research should be conducted to summarize the screening in different regions. Newborns born in our hospital from January 1, 2023, to December 31, 2023, were selected as the study participants, aiming to explore the application value of the “joint index” in neonatal CHD screening and to provide reference and basis for the early prevention and treatment of CHD in the neonatal period.

## Materials and methods

### Participants of the study

Neonates with postnatal age of 6–72 h delivered at the Department of Obstetrics and Gynaecology of Sichuan Provincial People’s Hospital from January 1, 2023, to December 31, 2023, were included as the study participants. Inclusion criteria were as follows: all live-born neonates during the period of the study, including mothers and infants in the same room and neonates transferred to the neonatal intensive care unit for medical reasons; and neonates whose informed consent was obtained from the legal guardians. The exclusion criteria were as follows: newborns who needed oxygen therapy or respiratory support for more than 3 days; those neonates who had discontinued oxygen therapy for less than 12 h; newborns who needed to maintain circulation with vasoactive drugs for a long time; those with severe infections (e.g., sepsis accompanied by shock and/or multiple organ failure)^9^; newborns suffering from severe neurological, digestive, and other systemic disorders (e.g., severe hypoxic-ischemic encephalopathy, stage III necrotizing enterocolitis)^10,11^; and children who were diagnosed with CHD using the prenatal ultrasound. A total of 3037 neonates were enrolled including 1565 males and 1472 females, and informed consent^12^ was obtained from the guardians. The Ethics Committee of our hospital approved the study (Approval No. QYYKJ-2025-24)^13^. The methods of this study were performed in accordance with the screening and management process of neonatal CHD in Chengdu.

### Research methodology

#### Grouping

“Combined indicator” means that newborns within 6–72 h of birth are screened for heart murmur auscultation and pulse oximetry, with a positive test for either or both of the two indicators. All newborns were screened by specially trained neonatal or obstetric clinicians. The newborns included in the study were categorized into the following three groups based on the screening results: a heart murmur-only group (murmur group), a positive pulse oximetry-only group (oximetry group), and a double-positive heart murmur and/or pulse oximetry group (combined group). A cardiac ultrasound was performed on all screen-positive newborns for clarification.

#### Procedures

**Fig. 1:**
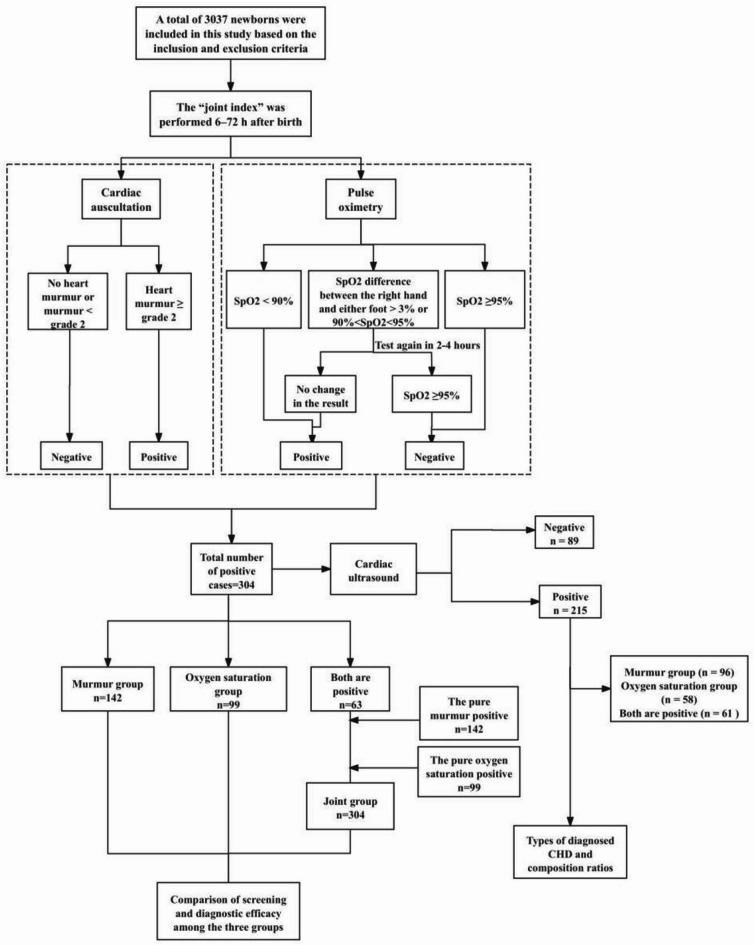
Screening Flowchart.

All neonates underwent pulse oximetry and cardiac auscultation screening, with those showing positive results in either cardiac murmur detection or pulse oximetry subsequently receiving cardiac ultrasonography (Fig. 1).

#### Heart murmur auscultation

A double-sided stethoscope was used for the infants. Cardiac auscultation included heart sounds, heart rate, rhythm, murmurs, and friction sounds. On the chest wall of the newborns in a quiet state, the stethoscope was placed, and auscultation was carried out. Auscultation was performed beginning from the mitral valve auscultation area, the strongest point of the apical beat, to the second intercostal space at the left edge of the sternum (the pulmonic valve area), the second intercostal space at the right edge of the sternum (the aortic valve area), the third intercostal space at the left edge of the sternum (the aortic valve’s second auscultation area), and the fourth and fifth intercostal spaces at the left edge of the sternum (the tricuspid valve area). The auscultation time for each area was a minimum of 10 s. A definite grade 2 or higher murmur was considered positive for screening.

#### Measurement of pulse oximetry

A reusable wrap-around test probe suitable for newborns was selected using the Welch Allyn Vital Signs Detector. The probe was cleaned before and after each use to avoid pressure sores and hypothermia burns, and pulse oxygen saturation (SpO2) was measured in the range of 1–100% with an accuracy of + 1%. The test probe was fixed on the cleaned palm of the right hand and the palm of either foot of the newborn. Data were recorded only when the heart rate displayed by the pulse oximetry tester matched the actual heart rate of the newborn, and the pulse oximetry value and signal waveform of the instrument were stabilized for at least 10 s, which was generally approximately 2 min. The pulse oximetry screening strategy was performed according to the 2021 American Academy of Pediatrics (AAP) guidelines for critical congenital heart disease detection^14^, emphasizing multi-site measurements (pre/post-ductal sites) and confirmatory repeat testing within 4 h. The protocol adhered to the following criteria: (1) SpO_2_ < 90% in the right hand or either foot; (2) SpO_2_ between 90% and 94% in the right hand or either foot on two consecutive measurements (interval: 2–4 h); (3) The SpO_2_ difference was > 3% between the right hand and either foot on two consecutive measurements (interval: 2–4 h).

#### Ultrasound examination of the heart

The positively screened newborns underwent a cardiac ultrasound. This study was conducted by senior physicians from the ultrasound department of a tertiary hospital to perform neonatal cardiac ultrasound examinations. All operators were certified with national qualifications for congenital heart disease screening and had over five years of experience in diagnosing CHD. Double-blind quality control was implemented, with the results being rechecked by physicians of the same qualifications. A diagnosis was confirmed only after a consensus was reached through double-blind rechecking, and the operation process strictly followed the standard section requirements of the “Technical Specifications for Neonatal Congenital Heart Disease Screening“^8^. PhilipsHD11 and HP4500 Doppler ultrasound diagnostic machines were used with a probe frequency of 3–7 MHz. To avoid errors in the instrumentation system, the same preset conditions were applied. The neonate was placed in a quiet position and a routine echocardiogram was performed with full exposure of the chest in the supine position to clarify the presence and type of CHD. Cardiac echocardiography can be used as the gold standard for CHD diagnosis. The following two conditions were followed up at 3 months of age: (1) unclosed patent foramen ovale: shunt bundle < 5 mm, peak velocity < 0.5 m/s, demonstrating a live flap, which is easily confused with an atrial septal defect (ASD) because of the bidirectional shunt, and those who had not yet been closed at 3 months of age; and (2) unclosed patent ductus arteriosus: those who had not yet been closed at 3 months of age were diagnosed with CHD.

#### Statistical methods

Data were analyzed using the SPSS 27.0 statistical software. Measurement data was expressed as ¯χ ± s, and the two groups were compared using the independent samples t-test. The count data was expressed as cases (percentage) (n [%]), and the χ2 test (including corrected χ 2 test) or Fisher’s exact probability method was used for comparison between groups. The statistical significance was set at *P* < 0.05.

## Results

### General information and results of the three screening groups

Of the total of 3037 newborns screened, there were 304 positive cases, with a positive screening rate of 10.00%, including 162 males (53.30%) and 142 females (46.70%). Gestational age and birth weight ranged from 33 to 41 weeks and 1920 to 4360 g, with an average of (38.6 ± 1.3) weeks and (3150 ± 411) g, respectively. A total of 215 cases with CHD were examined by cardiac ultrasound in the screen-positive cases, of which 142 cases were positive in the pure murmur group (murmur group), and 96 cases were confirmed positive by cardiac ultrasound results. The number of positive cases in the oxygen saturation alone group was 99, and the number of confirmed positive cases was 58. The number of positive cases in the combined group was 304, including 63 positive for both heart murmur and oxygen saturation. The total number of confirmed positive cases was 215 (see Table 1). No statistically significant differences were found in the sex composition, gestational age, and birth weight of the newborns in the three groups (see Table 2, *P* > 0.05).

**Table 1:** Results of screening for the three groups.

Cardiac ultrasound findings				
Grouping		Positive (*n* = 215)	Negative (*n* = 2822)	Total (*n* = 3037)
Murmur group	Positive Negative	96 119	46 2776	142 2895
Oxygen saturation group	Positive Negative	58 157	41 2781	99 2938
Joint group	Positive Negative	215 0	89 2733	304 2733

**Table 2:** The summary of the basic information for the three groups.

	Murmur group (*n* = 142)	Oxygen saturation group (*n* = 99)	Joint group (*n* = 304)	F/χ2	*P* value
Gender (cases Male: Female)	77:65	46:53	162:142	1.682	0.431
Gestational age (weeks)	38.7 ± 1.2	38.4 ± 1.5	38.6 ± 1.3	1.652	0.193
Birthweight (g)	3170 ± 380	3120 ± 447	3150 ± 411	0.482	0.618

### Comparison of screening and diagnostic efficacy among the three groups

The diagnostic sensitivity, accuracy, and negative predictive value(NPV)of the combined group were 100%, 97.07%, and 100%, respectively, using the results of cardiac ultrasonography as the gold standard, which were significantly higher than those of the cardiac murmur and oxygen saturation groups, and the difference was statistically significant (*P* < 0.01). When the results of the three groups were compared (*P* > 0.05), there was no statistically significant difference in the positive predictive value (PPV); however, a two-by-two comparison of the combined and oxygenation groups demonstrated that the PPV of the combined group was greater than that of the oxygenation group, and the difference was statistically significant (χ2 = 5.035; *P* < 0.05). The specificity of the combined group was 96.85%, which was lower than that of the murmur and oxygenation groups (*P* < 0.01) (Table 3).

**Table 3:** A comparison of diagnostic efficacy of the three groups (% [n/m])

Group	Sensitivity	Specificity	Accuracy	PPV	NPV
Murmur group	44.65	98.37	94.57	67.61	95.89
Oxygenation group	26.98	98.55	93.48	58.59^b^	94.66
Joint group	100	96.85	97.07	70.72^a^	100
χ2	254.947	24.243	43.638	5.033	140.735
*P*	< 0.01	< 0.01	< 0.01	0.081	< 0.01

### Types of diagnosed CHD and composition ratios

A total of 215 cases of CHD were diagnosed by cardiac ultrasonography, of which the top three types were as follows: atrial septal defect (ASD), ventricular septal defect (VSD), and Patent ductus arteriosus (PDA) in 73 (33.95%), 56 (26.05%), and 44 (20.47%) cases, respectively. Other congenital cardiovascular developmental anomalies included 3, 2, and 1 cases of permanent left superior vena cava, vagal right subclavian artery, and mirror right heart, respectively (Table 4). According to the grading of severity of CHD by Ewer^15^, Hoffman^16^, and other scholars, CHD requiring intervention before 1 year of age was categorized as severe in the present study. Of the 215 cases of diagnosed CHD, 26 cases of severe CHD were detected (including two of pulmonary stenosis (PS), two of tetralogy of Fallot (TOF), one of partial ectopic pulmonary venous drainage (PAPVC), one of transposition of the great arteries (TGA), seven of ASD combined with larger shunts and VSD with larger shunts, and 13 cases of severe preterm PDA), of which three cases were detected in the murmur group, nine in the oxygenation group, and a total of 14 cases of severe CHD were detected in cases with positive cardiac murmurs and oxygen saturation in the combined group (63 cases), with detection rates of 11.54%, 34.62%, and 53.83%, respectively, which were statistically different in the comparison of all three cases (χ2 = 10.500; *P* < 0.01).

**Table 4:** The composition of congenital cardiac anomalies in 215 cases.

Conditions	Cases counts	Percentage	Murmur	Oxygenation	Joint
		(%)	group	group	group
ASD	73	33.95	42	15	73
VSD	56	26.05	23	9	56
PDA	44	20.47	15	21	44
PDA + ASD	16	7.44	9	4	16
ASD + VSD	11	5.12	4	2	11
ASD + VSD + PDA	3	1.40	1	1	3
PS	2	0.93	0	0	2
TOF	2	0.93	0	2	2
PAPVC	1	0.46	0	1	1
TGA	1	0.46	0	1	1
Other anomalies	6	2.79	2	2	6
Total cases	215	100	96	58	215

## Discussion

CHD is one of the most common types of birth defects with complex etiology and ambiguous pathogenesis. Current research suggests its close association with a variety of factors, such as genetics, environment, and intrauterine infections^17^, thus making it the primary cause of neonatal and infant mortality^18^. CHD may present with a variety of serious complications such as pneumonia, pulmonary hypertension, heart failure, and shock, which threaten children’s health. Therefore, early screening for CHD is particularly important. Echocardiography is the gold standard for clinical diagnosis of CHD; however, because of the difficulty and high cost of operation, its promotion in grassroots hospitals is challenging. In addition, there are varying levels of knowledge and diagnosis of CHD among medical personnel in midwifery institutions in China. Up to 71% of asymptomatic critically ill patients with CHD are not detected before maternal discharge^19^. Therefore, a more economical, effective, and convenient approach for early detection of CHD is anticipated, based on which, this study explores the value of heart murmur combined with pulse oximetry in the diagnosis of CHD, and provides an important auxiliary clinical tool to detect CHD as early as possible.

Heart murmur auscultation is an important component of the physical examination of the heart and an effective screening tool for early detection of neonatal CHD. Most CHDs consist of a heart murmur^20^. However, early neonatal cardiovascular anatomy, pressure, blood flow, and vascular resistance in the physical and pulmonary circulation have undergone drastic changes because of the transformation of fetal to neonatal circulation, resulting in the emergence of a heart murmur, called a physiologic murmur. Therefore, the presence of a heart murmur in the neonate does not necessarily indicate CHD. Moreover, some of the heart murmurs are atypical or even absent in CHD because of the small pressure difference between the left and right hemispheres of the heart in the neonatal period. Therefore, a risk of underdiagnosis or misdiagnosis of CHD by heart murmur screening alone exists. In this study, the sensitivity, specificity, and accuracy of CHD screening by heart murmur alone were 44.65%, 98.37%, and 94.57%, respectively, and the specificity was improved than that of previous studies^21^, which is consistent with the recent study by Huang et al.^22^.The training of medical personnel in cardiac auscultation has been increased because the Department of Maternity and Childhood of the National Health Commission officially launched the “neonatal congenital heart disease screening” program, promoting neonatal CHD screening nationwide. In addition, there is a significant increase in the awareness and attention of medical personnel to neonatal CHD, which has reduced the risk of misdiagnosis and underdiagnosis of cardiac auscultation to some extent.

As a non-invasive and simple test neonatal SpO2 measurement is commonly used in the screening of neonatal CHD for early identification of clinically asymptomatic mild-to-moderate hypoxemia. It is especially important for the diagnosis of severe CHD^23^. In this study, the sensitivity, specificity, and accuracy of SpO2 in screening for CHD were 26.98%, 98.55%, and 93.48%, respectively, indicating high specificity and accuracy but low sensitivity, a result confirmed in previous reports^22,24^. The small fractional flow rate of some types of CHD, the absence of significant hypoxemia, or the presence of catheter-dependent CHD may all lead to a missed diagnosis. In the present study, the sensitivity, accuracy, and negative predictive value of neonatal CHD screening using SpO2 and heart murmur as a combined index were significantly improved. The results were similar in positive predictive value, while the specificity in the combined group was lower than those in the murmur and oxygen saturation groups Considering that this study was a single-center study and the study unit was a critical care center for pregnant women with high-risk neonates, the specificity of screening may be interfered with by infectious and pulmonary diseases in the neonatal period. Therefore, future data from larger samples and multiple centers are needed to validate the results. Additionally, of the 26 cases of severe CHD identified in this study, 14 were detected in the combined group, and the detection rate was as follows: combined group > oxygenation group > murmur group. Therefore, the confirmation rate of early neonatal CHD screening can be improved, the rate of missed diagnosis can be reduced, and detection rate of severe CHD may be increased using the “combined indicator” screening.

It is worth mentioning that the two cases of TOF, one case of PAPVC, and one case of TGA found in this study were detected by the oxygen saturation alone group, depicting that although SpO2 has a low sensitivity in screening for CHD, it is more important in screening for critical and rare CHD. The auscultation of heart murmurs is more capable of screening for common, mild CHD; therefore, the two may play a complementary role as a combined indicator for screening CHD. Additionally, with the introduction and application of the “integrated” model of prenatal and postnatal care for congenital heart disease^25^, fetal echocardiography has an important clinical role as the critical aid for prenatal diagnosis of CHD, and fetal CHD, especially severe CHD, prenatally. However, the four cases of severe CHD were not diagnosed prenatally because of regional and family financial constraints. This demonstrates the necessity of prenatal diagnosis in the early diagnosis of neonatal CHD. In contrast, this also highlights the importance of SpO2 combined with heart murmur as a cost-effective and convenient screening tool in the detection of severe CHD in neonates.

This study needs to point out that due to the characteristics of the early circulatory physiological transition in newborns, the pulmonary vascular resistance has not yet decreased, and the pressure difference between the pulmonary and systemic circulation has not been significantly imbalanced. This may lead to the early symptoms of left-to-right shunt congenital heart disease (such as heart murmur and abnormal blood oxygen saturation) being atypical. Using “combined indicators” for screening may have a risk of missed diagnosis. Given this, neonatal screening needs to be combined with systematic follow-up after birth. As emphasized by the AHA guidelines (2019), the ultimate goal of congenital heart disease screening is to build a seamless connection system of “screening - diagnosis - intervention”^26^. The combined indicator screening serves as a primary early warning tool, and its core value lies in quickly identifying high-risk populations. Subsequent multimodal follow-up (such as cardiac specialist physical examination, dynamic observation of heart murmur changes, and timely echocardiography re-examination) can effectively compensate for its limitations. Only through the combined effect of both, the full-cycle management of congenital heart disease, from the perinatal period to childhood, can be achieved.

In conclusion, our study demonstrates that the newborns can be screened for CHD using heart murmur auscultation combined with pulse oximetry that is a convenient, sensitive, and accurate method. It can be utilized as an important tool for early clinical screening for CHD.

## Data Availability

The datasets generated during and/or analyzed during the current study are available from the corresponding author on reasonable request.
